# Profiling of Oral Bacterial Communities

**DOI:** 10.1177/0022034520914594

**Published:** 2020-04-14

**Authors:** W.G. Wade, E.M. Prosdocimi

**Affiliations:** 1 Centre for Host-Microbiome Interactions, Faculty of Dentistry, Oral & Craniofacial Sciences, King’s College London, London, UK; 2 Forsyth Institute, Cambridge, MA, USA

**Keywords:** microbiome, caries, periodontitis, gingivitis, ecology, dentistry

## Abstract

The profiling of bacterial communities by the sequencing of housekeeping genes such as that encoding the small subunit ribosomal RNA has revealed the extensive diversity of bacterial life on earth. Standard protocols have been developed and are widely used for this application, but individual habitats may require modification of methods. This review discusses the sequencing and analysis methods most appropriate for the study of the bacterial component of the human oral microbiota. If possible, DNA should be extracted from samples soon after collection. If samples have to be stored for practical reasons, precautions to avoid DNA degradation on freezing should be taken. A critical aspect of profiling oral bacterial communities is the choice of region of the 16S rRNA gene for sequencing. The V1-V2 region provides the best discrimination between species of the genus *Streptococcus*, the most common genus in the mouth and important in health and disease. The MiSeq platform is most commonly used for sequencing, but long-read technologies are now becoming available that should improve the resolution of analyses. There are a variety of well-established data analysis pipelines available, including mothur and QIIME, which identify sequence reads as phylotypes by comparing them to reference data sets or grouping them into operational taxonomic units. DADA2 has improved sequence error correction capabilities and resolves reads to unique variants. Two curated oral 16S rRNA databases are available: HOMD and CORE. Expert interpretation of community profiles is required, both to detect the presence of contaminating DNA, which is commonly present in the reagents used in analysis, and to differentiate oral and nonoral bacteria and determine the significance of findings. Despite advances in shotgun whole-genome metagenomic methods, oral bacterial community profiling via 16S rRNA sequence analysis remains a valuable technique for the characterization of oral bacterial populations.

## Introduction

The characterization of the human oral bacterial community by targeted amplification and sequencing of the 16S ribosomal RNA gene is now well established and has been used as the basis for the Human Oral Microbiome Database (Dewhirst et al. 2010). The use of next-generation sequencing methods has led to a step change in the numbers of sequence reads generated, giving vastly improved depth of coverage to the analysis. These methods have enabled the diversity of bacteria and archaea found in the human mouth to be comprehensively catalogued and associations made between specific taxa and health and disease states.

The aim of this review is to provide an overview of oral bacterial community profiling and discuss some practical considerations, particularly where methods suitable for oral studies differ from those commonly used for investigations of other body sites and/or the environment. A more detailed and general discussion of the methodological options for microbiome studies can be found elsewhere (Pollock et al. 2018).

## Value and Limitations of Community Profiling Analyses

Community profiling enables the comparison of the composition of the microbiota in different experimental groups, for example, cases of a disease versus controls, samples collected from different body sites, changes over time, and the effect of treatment. The standard methodology does not detect nonbacterial microorganisms. Polymerase chain reaction (PCR) primers can be modified to include detection of Archaea or designed specifically for that domain. Fungi and protozoa can be studied by using 18S rRNA genes, and internal spacer (ITS) regions are frequently used to identify fungi (Ghannoum et al. 2010). Characterization of the oral virome is an expanding area, but most viruses found in the mouth have yet to be classified, and the majority appear to be bacteriophages (Pride et al. 2012).

The major weakness of profiling methods is they do not quantify bacterial load. The primary output will be a table giving the number of sequences that correspond to bacterial taxa or operational taxonomic units (OTU) for each sample, which is typically presented as relative abundance. This is an important limitation, particularly for treatment studies. Successful treatment of an infection may result in significant reductions in bacterial numbers. The proportions of taxa in the posttreatment samples may therefore not be of biological relevance. Samples can be spiked with known amounts of a reference bacterium to give some measure of quantitation (Stammler et al. 2016). Where absolute numbers of an individual known species are required, quantitative PCR should be used (Maeda et al. 2003).

**Figure 1.**
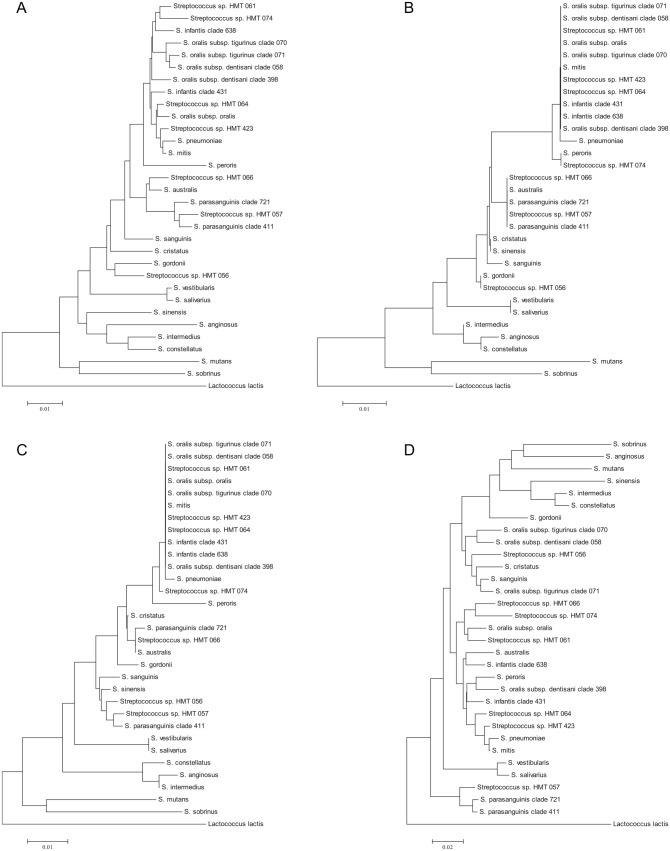
Phylogenetic trees based on 16S rRNA gene sequence comparisons showing relationships between oral streptococcal species for different regions of the gene. The trees were reconstructed using the neighbor-joining method from a distance matrix constructed from aligned sequences using the Jukes-Cantor correction. (**A**) A total of 1343 unambiguously aligned bases over the full length of the gene. (**B**) V4 region, 252 bases. (**C**) V3-V4 region, 427 bases. (**D**) V1-V2 region, 326 bases.

Oral bacterial community profiling reveals which bacteria are present but not how they are interacting with the host and other microorganisms. Techniques have been developed to investigate microbial function including metagenomics, which describes the functional genetic potential within samples (Alcaraz et al. 2012), and metatranscriptomics, which investigates the genes being actively transcribed at sites at particular times (Duran-Pinedo et al. 2014). Bacterial genomes can be assembled from raw shotgun metagenomic data to construct metagenome-assembled genomes (Bowers et al. 2017). These are of value for the reconstruction of metagenomic pathways within organisms and the prediction of bacterial-bacterial and bacterial-host interactions. Accurate assembly can be compromised, however, in complex bacterial communities that include closely related taxa, such as the mouth. For example, the genus *Streptococcus* includes a large number of species, many of which cluster together in closely related groups (Fig. 1). Many streptococci are naturally competent and share DNA, further confusing species boundaries (Hanage et al. 2005; Fraser et al. 2007). Metagenomic pathways assembled from oral samples are thus likely to be composite genomes made up of different species (Shaiber and Eren 2019). For this reason, community profiling allows a description of the species present in a sample at higher resolution than current metagenomic methods. Thus, although 16S rRNA gene community profiling is sometimes regarded as a dated method that has been superseded by shotgun metagenomic analyses, a recent comparison showed that it was a valuable method of bacterial community characterization (Rausch et al. 2019) and is considerably more cost-effective.

## Practical Considerations for Oral Bacterial Community Profiling

**Figure 2.**
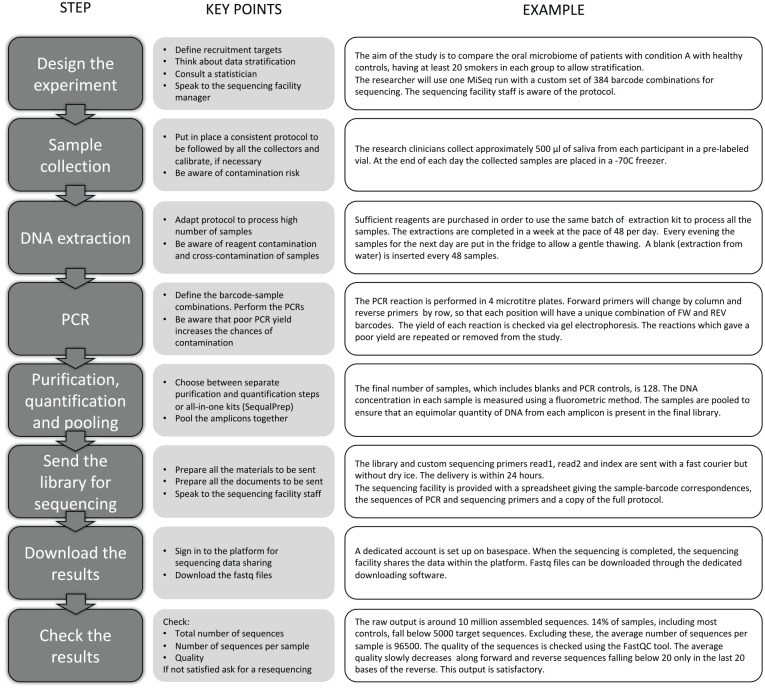
Key steps and considerations for the design and performance of oral bacterial community profiling studies.

Figure 2 shows the stages involved in sample collection and processing for an oral microbiome study.

### Study Design

A statistician should be consulted at the design stage. For oral microbiome investigations, the numbers of samples to be included is of particular importance. The oral microbiome is highly variable between individuals and is also stable and markedly resilient to change (Zaura et al. 2015; Rosier et al. 2018). Thus, to demonstrate significant differences between individuals with differing disease states or to see the effect of a treatment, substantial numbers of subjects may be required. Power calculation methods for microbiome studies are now available (Kelly et al. 2015), and the stratification of subjects is often of value in detecting differences between groups (Mattiello et al. 2016).

It is critical to collect clinical metadata appropriate for the study. As mentioned above, the individual has the strongest influence on oral bacterial community composition, followed by oral disease status. In particular, the presence of active caries, the extent of gingival inflammation, and the presence and severity of periodontitis should be recorded. The need for appropriate clinical metadata is often a limiting factor in study feasibility.

### Sample Collection

The prime consideration in sample collection is to ensure that sufficient biomass is collected to give a good bacterial DNA yield. Low yields can lead to the emergence of contaminating DNA in libraries. In practice, useable oral samples are relatively easy to obtain. Just 0.25 mL of saliva or plaque collected from 1 or more teeth provides enough DNA for good profiling. The sample collected should also be appropriate to the research question. Saliva was once thought to represent all of the bacteria found on oral surfaces, but it is actually strongly biased toward the tongue and palate communities (Segata et al. 2012). Sampling mucosal sites can be challenging because the bacteria of interest may be firmly attached or within the tissues and present at levels lower than in the saliva bathing the site. Rinsing the mouth with sterile saline and drying the site with sterile gauze is advised, before sample collection with a swab or gentle scraping.

### Sample Storage and Processing

If possible, DNA should be extracted from samples on the day of collection and stored at −80°C. However, this is not time or labor efficient if a study’s recruitment rate is low. It has been shown that samples can be stored frozen without grossly affecting the proportions of OTUs detected (Lauber et al. 2010). It is important, however, to include a cryoprotectant to prevent damage done to sample DNA by the formation of ice crystals (McKain et al. 2013). A number of suitable storage media are commercially available. When samples are later used, it is important that they are processed at the same time because significant batch effects have been seen in microbiome studies (Weiss et al. 2014).

Choice of DNA extraction method is a potential source of bias. Because of their thick peptidoglycan layers, gram-positive bacteria are more difficult to lyse than gram-negative bacteria, and the use of an enzymatic treatment such as lysozyme or physical disruption with bead beating is recommended. Comparisons of different DNA extraction methods for oral samples have, however, yielded equivocal results, with, for example, one study finding significant differences between methods (Abusleme et al. 2014) but another failing to do so (Rosenbaum et al. 2019).

### Choice of Sequencing Platform

The most widely used sequencing platform for bacterial community profiling is currently the Illumina MiSeq. Although the Illumina 600 cycle kit yields 2 × 300-bp paired reads, it is not advisable to use this to sequence a 500-bp fragment, for example, the 16S rRNA V1-V3 region. This is because the quality of Illumina sequences declines markedly toward the ends of reads, and with a short overlap between paired reads, poor-quality assemblies will result that will manifest themselves as spurious diversity in the data set (Kozich et al. 2013) or, if adequate quality filtering is applied, a high proportion of assembled sequences will be removed, reducing the depth of coverage.

Amplicon sequencing protocols are available for 2 long-read sequencing technologies, PacBio and Oxford Nanopore, which enable the full length of the 16S rRNA gene to be sequenced (Calus et al. 2018; Callahan et al. 2019). The relevant Nanopore kit enables up to 12 barcodes, and therefore samples, to be sequenced. Although this is far lower than the MiSeq protocols, the analysis is rapid and could be useful if quick results are required.

### Which Region of the 16S rRNA Gene?

As discussed above, the MiSeq platform generates reliable data of up to about 350 bp. The region of the 16S rRNA gene to use is therefore critically important. The most widely used protocols have targeted the V4 or V3-V4 regions, which provide profiles representative of diverse communities at the genus level. The microbiota found in many habitats is poorly characterized, with a high proportion of unnamed species level taxa. The human mouth bacterial community, by comparison, is relatively well characterized at the species level, and even where species-level taxa are unnamed, they have been given reference taxa numbers in the Human Oral Microbiome Database (www.homd.org). The functional capability of oral bacteria is also well known, and different species within genera have very different biological properties. For example, *Streptococcus mutans* and related species are associated with dental caries (Gibbons and van Houte 1975), while *Streptococcus salivarius* is health associated and has been proposed for use as a probiotic (Burton et al. 2011) and the *Streptococcus anginosus* group is associated with a number of systemic infections (Fazili et al. 2017). Figure 1 shows phylogenetic trees for oral streptococci prepared from alignments corresponding to amplicons obtained with primers for the V1-V2, V3-V4, V4, and the near full-length gene. It can be seen that the V4 and V3-V4 regions differentiate oral streptococci poorly, while analysis of the V1-V2 region is capable of identifying most streptococci to species level and is thus at present recommended for the study of oral samples (Cabral et al. 2017). Further work should be performed, however, to determine the utility of different regions of the gene for differentiation of all oral bacterial species. The template-specific primers recommended for V1-V2 are the YM modification of 27F (Frank et al. 2008): 5′-AGAGTTTGATYMTGGCTCAG-3′ and 338R: 5′-TGCTGCCTCCCGTAGRAGT-3′, which can be incorporated into fusion primers with appropriate adapters and barcodes (Kozich et al. 2013). Because next-generation sequencing methods are prone to high sequence error rates, proofreading DNA polymerases should be used (Gohl et al. 2016).

### Sample Indexing

Individual PCR primers can be labeled by adding a barcode, but this is expensive because a different labeled primer is required for each sample. Dual indexing, in which the amplicons from each sample are labeled at both ends, is more cost-effective. This can be done in a single-stage process in microplate format in which, for each plate, 8 forward primer barcodes are combined with 12 reverse barcodes to give 96 combinations. If second sets of forward and reverse barcodes are prepared, and all combinations are used, 384 samples can be amplified and indexed, giving a potential depth of coverage of 10,000 assembled paired reads per sample. In practice, the number of reads obtained from different samples is unequal, despite careful equimolar pooling, and 5,000 reads per sample is realistic and gives a good level of coverage, with Good’s coverage values of 98% or higher. An alternative method of indexing is the 2-stage method, as used in the Illumina Nextera kit. One set of primary PCR primers with adapters on each primer is used to amplify the target region in the samples. The amplicons are then labeled in a second PCR with primers specific for the adapters.

### Purification and Pooling of Amplicons

The amplicons from each sample are purified to remove excess primer and incomplete amplicons. The amplicons from each sample are then quantified and mixed together in equal amounts for sequencing. The concentration of each product is then adjusted before pooling. Finally, if multiple plates have been used, the pool from each plate is quantified and concentrations adjusted before mixing to create a final pool that is submitted for sequencing.

### Controls

A mixed community control should be used to demonstrate that the PCR conditions used yield profiles that adequately represent the community. Mixtures of genomic DNAs from different bacterial species at known concentrations are commercially available.

Most DNA extraction and PCR reagents are contaminated with low levels of DNA (de Goffau et al. 2018). If the sample size is large enough, this is not a problem because the contaminating DNA will be present at only low levels compared with the DNA extracted from the sample. It has been shown that when DNA from a pure culture of a bacterial strain is diluted, the proportion of contaminants seen progressively rises (Salter et al. 2014). The contaminating organisms are typically those found in the environment, and their DNA is often present in tap water. Typical contaminating genera include *Acinetobacter, Bradyrhizobium, Comamonas, Janthinobacterium, Methylo-bacterium, Pseudomonas, Ralstonia, Sphingomonas, Steno-trophomonas*, and *Xanthomonas* (Tanner et al. 1998; Munson et al. 2002; Salter et al. 2014). The finding of these or related genera in libraries prepared from oral samples should be regarded as suspicious. The detection of Proteobacteria, in particular, in oral samples can be genuine, however. Patients with dry mouths, immunodeficiency, oral cancer, and other conditions can be become colonized with nonoral bacteria, particularly *Enterobacteriaceae* and *Pseudomonas* and related genera (Fernandes-Naglik et al. 2001). Similarly, a study of the oral microbiota in noma, an aggressive tissue-destructive disease, in Africa found high proportions of these types of bacteria (Paster et al. 2002). Careful interpretation of microbiomic data is therefore always required.

A negative control, for example, sterile DNA-free water, should therefore be included. The removal of major contaminant OTUs can be done manually through a careful inspection of the taxonomy table. Alternatively, the R package decontam can be used to identify and remove contaminants (Davis et al. 2017).

## Data Analysis

A number of user-friendly pipelines for the analysis of 16S rRNA gene sequence data are available. The use of default settings in these pipelines will generate draft summary tables and figures from a data set within a day in most cases. Accurate and informative analysis, however, will require that settings be modified to suit the type of data being analyzed and the research questions being asked. Most genome centers now have bioinformaticians familiar with the standard pipelines who can offer useful advice or perform the analyses, but specialist interpretative advice from an experienced oral microbiologist for oral samples is likely to be needed.

**Figure 3.**
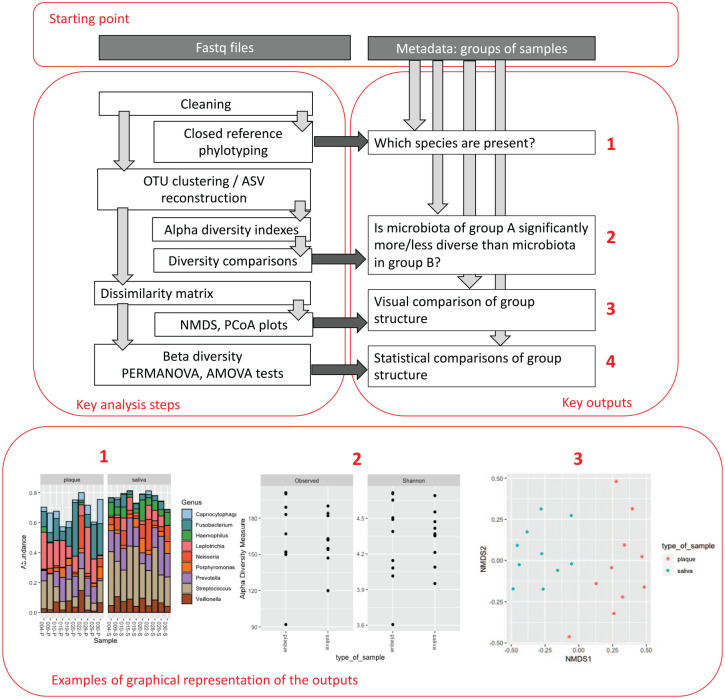
Overview of computational analysis of bacterial community profiling data.

An overview of the analysis process is shown in Figure 3. Analyses can be run on desktop computers, but a fast processor and large amount of RAM will be valuable. For larger data sets, a high-performance computing cluster will be required. The most commonly used analysis pipelines are QIIME 2 (Bolyen et al. 2019) and mothur (Schloss et al. 2009). Both pipelines are well documented and allow the user to choose a variety of data-filtering and -analysis methods. The default options may not be suitable for all applications, and new users are strongly recommended to seek expert advice.

For MiSeq data, the genome center will provide 2 FASTQ files for each sample: the forward and reverse reads. These will be filtered for length and quality and assembled. Sequences will then either be classified by comparison to a reference data set, a process known as phylotyping, or grouped into OTUs either in a closed way by again comparing to a reference data set or de novo, in which sequences are grouped purely on their similarity. This is typically performed at a sequence identity level of 97%, which was considered to equate to a “species”-level identification. It is known, however, that many validly established related oral species have very different biological properties but share greater than 97% 16S rRNA gene sequence identity. For this reason, it is recommended that OTUs for oral studies be constructed at 98.5% or 99%. The distribution of the OTUs thus formed among samples is then displayed in an OTU or shared table.

A potential source of bias is that bacterial species vary in the number of copies of the ribosomal RNA operon included in their chromosome. Databases of rRNA operon copy number have been constructed (Stoddard et al. 2015), and software tools are available to correct data sets for copy number (Angly et al. 2014). The rRNA operon copy number remains unknown, however, for perhaps the majority of oral taxa, limiting the value of such corrections. In practice, however, because most analytical comparisons are of the relative proportions of taxa between samples, the effect of copy number bias is limited (Pollock et al. 2018).

### The OTU Table

The starting point for further analysis is a table showing the numbers of each OTU by sample. Such a table can have hundreds of rows (depending on the size of the study) and thousands of columns (depending on the clustering parameters chosen). The most common goal of a microbiome study is to determine if the microbiome in 2 groups of samples differs significantly. The microbiome in a sample is represented by the relative abundances of all the single OTUs (i.e., all the columns in the table), each of them being a single variable. This goal can thus be achieved only by means of a multivariate statistical analysis, capable of taking into account many different variables at the same time. The distribution of the number of sequences of a given OTU in the samples is not normal, and especially for rare OTUs can contain many zeroes. To obtain a list of the species in the sample, a consensus identification of the sequences within each OTU can be obtained. While this method allows a straightforward comparison between classification and beta-diversity analyses, it may be misleading. OTUs often include multiple species, and species can be found in multiple OTUs. Because of this, and the inability of partial 16S rRNA gene sequences to resolve to species level, many authors classify OTUs to genus only. An alternative is to run a parallel phylotyping analysis, in which each single sequence is compared with the database.

Whichever analysis pipeline is chosen, the use of a curated database greatly improves the quality of the analysis. There are 2 high-quality curated oral bacterial 16S rRNA databases available: HOMD (Chen et al. 2010) and CORE (Griffen et al. 2011). The typical analysis starts with the estimate of the diversity within each sample, or alpha diversity, using ecological indexes such as the Shannon index or Inverse Simpson index. To verify if the diversity is significantly different in given groups of samples, it is appropriate to compare the mean value of the Shannon or Inverse Simpson indexes using the Wilcoxon rank-sum test, which does not assume a normal distribution of the variable.

The core of the analysis is beta diversity comparisons: at this step, the whole microbiome in given groups of samples is compared. The traditional approach to multivariate analysis involves the following steps: (1) creating a dissimilarity matrix, compiled of values that represent the difference between each possible couple of samples in terms of microbiome (popular metrics to calculate these distance are Bray-Curtis dissimilarity or theta-YC); (2) using the dissimilarity matrix to assess if the dissimilarity values within groups of samples are significantly shorter than distances among groups (the most widely used statistical tests to achieve this are permutational analysis of variance; Anderson 2001) or analysis of molecular variance (Excoffier et al. 1992). These tests allow the researcher to conclude, for example, that the microbiome of case studies is or is not significantly different from the microbiome of controls. The dissimilarity matrix can also be used to represent graphically the distances between samples through an nonmetric multidimensional scaling or principal coordinate analysis plot.

#### Normalization

There is a debate about how to normalize the data in the OTU table prior to the analysis. Random subsampling of even numbers of sequences per sample and transformation to proportions have been criticized (McMurdie and Holmes 2014). The arguments against the use of proportions are strong where the number of sequences per sample varies by 2 or more orders of magnitude and the number of samples is limited, as sometimes happens with environmental studies. A well-designed oral study will contain hundreds of samples, and by paying attention to the DNA quantification and pooling steps, it is possible to obtain a number of sequences per sample within the same order of magnitude. In these conditions, the results will likely be consistent whether or not a transformation is used.

A typical feature of oral microbiome data is a highly positively skewed distribution. A few species are responsible for the majority of the sequences, but the majority of species are very rare and absent from most samples. Some of the transformations, such as random subsampling, will affect these species the most, as they are likely to disappear from some samples. Although the importance of these rare members should not be underestimated, and they may include species with potent biological properties, their numerical presence will be unlikely to influence the outcome of the main beta diversity analysis.

### Alternatives to OTU Clustering

Even the most accurate sequencer introduces a certain amount of error in its reads, which will lead to inflated estimates of diversity. The most recently introduced analysis tools, however, make an attempt at correcting the error component. For example, the DADA2 pipeline includes an algorithm that aims to reconstruct the original sequence variants in the data set (Callahan et al. 2016). Instead of an OTU table, it will produce a table of amplicon sequence variants whose number is usually significantly lower than OTUs. The DADA2 algorithm can be used within the QIIME suite or as a separate R package. R users will find the latter solution very convenient, with the results that can be easily handled with the package phyloseq (McMurdie and Holmes 2013) or further analysed using DeSeq (see the following paragraph).

### Biomarker Discovery

Whenever a significant difference is found between experimental groups, the next step is to identify the OTUs responsible for the difference observed. LeFse can be used to find significant proportional differences between the groups (Segata et al. 2011) An alternative method is DeSeq2 (Love et al. 2014). Originally developed to analyze transcriptomic outputs, this R package takes the whole untransformed OTU table, on the assumption that the binomial model is more appropriate than any normalization (McMurdie and Holmes 2014). An example of the use of DeSeq with microbiome data can be found at https://bioconductor.org/packages/devel/bioc/vignettes/phyloseq/inst/doc/phyloseq-mixture-models.html.

### Resolution

For some taxa, higher-resolution analysis is required. For example, the 16S rRNA gene sequence of the human pathogen *Streptococcus pneumoniae* is virtually identical to that of the oral commensal *Streptococcus mitis*. Careful examination of aligned sequences, however, revealed that a cytosine at position 203 was present in all of 440 strains of *S. pneumoniae* but was replaced by an adenosine residue in all strains of other species of the mitis group streptococci (Scholz et al. 2012). This single base can therefore be used to identify strains of *S. pneumoniae*. The systematic analysis of sequence data to find small but consistent differences between strains is known as oligotyping. Oligotyping is based on the principle that while the sequencing error appears randomly in the sequence, the phylogenetically significant differences are found only in specific positions. Oligotyping and its automated version, called maximum entropy decomposition, can be used as an alternative to OTU clustering but can also be applied to single OTU-level groups of sequences, to obtain a finer discrimination to species or even strain level (Eren et al. 2013, 2015). A strain-level oligotype of *Streptococcus salivarius* present in saliva was recently shown to be specifically associated with Crohn’s disease and orofacial granulomatosis (Goel et al. 2019).

## Concluding Comments

16S rRNA-based bacterial community profiling via next-generation sequencing is currently the standard procedure to determine the composition of complex bacterial communities. Sequence costs are falling all the time, and the emergence of long-read technologies will transform shotgun metagenomic methods and enable communities to be profiled to a depth equivalent to that now possible with amplicon-based methods. Determining the composition from metagenomic data, however, relies on comparison with database sequences. The marked variability of genome composition between strains of the same species means that for whole-genome fragment comparisons to be accurate, a sufficient number of reference genomes for each species needs to be available. This is particularly difficult to achieve for those species that remain refractory to culture. For now, then, there will remain a place for 16S rRNA gene-based analyses, which are particularly effective for the characterization of the oral microbiota, thanks to the highly curated databases and extensive literature available.

## Author Contributions

W.G. Wade and E.M. Prosdocimi, contributed to conception and design, drafted and critically revised the manuscript. All authors gave final approval and agree to be accountable for all aspects of the work.
